# Adsorptive mutation and N-linked glycosylation modulate influenza virus antigenicity and fitness

**DOI:** 10.1080/22221751.2020.1850180

**Published:** 2020-12-14

**Authors:** Joshua E. Sealy, Thomas P. Peacock, Jean-Remy Sadeyen, Pengxiang Chang, Holly J. Everest, Sushant Bhat, Munir Iqbal

**Affiliations:** a Avian Influenza, The Pirbright Institute, Woking, UK; b Department of Infectious Diseases, Imperial College London, London, UK; c Nuffield Department of Medicine, University of Oxford, Oxford, UK

**Keywords:** Influenza, antigenic drift, avidity, receptor binding, glycosylation, virus evolution

## Abstract

Influenza viruses have an error-prone polymerase complex that facilitates a mutagenic environment. Antigenic mutants swiftly arise from this environment with the capacity to persist in both humans and economically important livestock even in the face of vaccination. Furthermore, influenza viruses can adjust the antigenicity of the haemagglutinin (HA) protein, the primary influenza immunogen, using one of four molecular mechanisms. Two prominent mechanisms are: (1) enhancing binding avidity of HA toward cellular receptors to outcompete antibody binding and (2) amino acid substitutions that introduce an N-linked glycan on HA that sterically block antibody binding. In this study we investigate the impact that adsorptive mutation and N-linked glycosylation have on receptor-binding, viral fitness, and antigenicity. We utilize the H9N2 A/chicken/Pakistan/SKP-827/16 virus which naturally contains HA residue T180 that we have previously shown to be an adsorptive mutant relative to virus with T180A. We find that the addition of N-linked glycans can be beneficial or deleterious to virus replication depending on the background receptor binding avidity. We also find that in some cases, an N-linked glycan can trump the effect of an avidity enhancing substitution with respect to antigenicity. Taken together these data shed light on a potential route to the generation of a virus which is “fit” and able to overcome vaccine pressure.

## Introduction

Influenza viruses are negative-sense segmented RNA viruses responsible for highly infectious respiratory disease in humans and variable pathology in important hosts such as aquatic birds and poultry, ranging from asymptomatic to fatal [1]. The infectious cycle of influenza begins with the adsorption of virus to host cell, facilitated by virus haemagglutinin (HA) binding to terminal sialic acid moieties of glycans on the surface of the cell [2]. A key component of the HA glycoprotein is the receptor binding site (RBS), a shallow pocket formed by amino acid side chains of conserved residues in the HA head [3]. Protective anti-HA antibodies elicited in response to influenza infection or vaccination are predominantly directed toward the RBS to inhibit virus adsorption and neutralize infection [4]. Despite vaccination, influenza viruses continuously circulate in humans and economically important animal hosts.

Antigenic drift is the accumulation of mutations in HA that enables evasion of pre-existing anti-HA antibodies induced by natural infection or vaccination. Influenza viruses have at their disposal several mechanisms for driving antigenic drift. The simplest mechanism involves an amino acid substitution that directly changes the biophysical property of a cognate epitope e.g. its shape or charge, which leads directly to diminished antibody binding [5]. A second, less common mechanism involves an amino acid substitution distal to a cognate epitope which diminishes antibody binding by an allosteric effect. This distal mutation reduces the flexibility of the globular domains of the HA1 trimer thus rendering an epitope inaccessible to antibody binding [6]. A third mechanism involves amino acid mutations that introduce an N-linked glycosylation motif (Asparagine-X-Serine/Threonine, where X is any amino acid other than proline) to the HA protein, with the resultant glycan acting to shield epitopes from antibody binding [7]. The fourth and final mechanism involves amino acid substitutions around the RBS which increase HA receptor binding avidity such that the binding equilibrium between HA and a neutralizing antibody is shifted in favour of the interaction between HA and SA [8–11].

For a single mutation to significantly alter antigenicity it must impose a radical change to the biophysical property of a virus; single mutations typically do this by introducing/removing an N-linked glycosylation motif, or by altering receptor binding avidity [10]. Alone, such mutations can be pleiotropic and induce a fitness cost in the absence of compensatory mutation [11–14]. Studies in the Yewdell lab described an immune escape virus with mutations that increased receptor binding avidity; however, this immune escape virus was found to require compensatory mutation to achieve viral fitness. The compensatory mutation introduced an N-linked glycan that restored receptor binding avidity to wild-type PR8 levels but retained antigenic divergence [13,15].

Previously, we characterized the impact of an adsorptive mutation in H9HA which impacted antigenicity, receptor binding and viral fitness [11]. Briefly, amino acid substitutions from alanine (A) to threonine (T) or valine (V) at HA residue 180 (mature H9 numbering used throughout) facilitated escape from chicken polyclonal antisera but imposed a fitness cost. This immune escape was due to changes in receptor binding avidity whereby T180 showed increased binding avidity compared to A180, and V180 showed increased binding avidity compared to A180 or T180 (from here on A180, T180 and V180 will be referred to as low, high, and highest avidity mutations, respectively). Due to the attenuating impact of increasing receptor binding avidity, we wanted to investigate the ability of N-linked glycosylation to compensate for avidity mutations. We found that the addition of an N-linked glycan to the HA protein is context dependent whereby a low avidity virus can be attenuated by the addition of an N-linked glycan, or in contrast, a high avidity virus can have improved viral fitness by the addition of an N-linked glycan. Furthermore, the addition of an N-linked glycan to a high avidity virus can lead to continued antigenic divergence.

## Materials and methods

### Cells and eggs

Chicken Kidney (CK) cells were prepared at the Pirbright institute three days prior to use in virus replication assays in 6-well plates as previously described [16] and maintained in CK cell media comprised of Eagle’s Minimum Essential Medium (EMEM) (Sigma-Aldrich) supplemented with 2 mM L-glutamine, 10% Bovine Serum Albumin (BSA), 1% penicillin–streptomycin and 10% tryptose phosphate broth. Madin-Darby Canine Kidney (MDCK) and Human Embryonic Kidney (HEK) 293 T cells were aseptically maintained in Dulbecco's modified Eagle medium (DMEM) (Sigma-Aldrich) supplemented with 10% fetal bovine serum (FBS) at 37°C in 5% CO_2_ without the addition of antibiotics. Virus was grown in 10-day old specific-pathogen-free (SPF) embryonated hens’ eggs and harvested at 48 h post-inoculation.

### Recombinant viruses and site-directed mutagenesis

Reverse-genetics (RG) viruses were generated from co-transfected bidirectional plasmids (pHW2000 or pCAGGs) as described previously [17,18]. Viruses were rescued with the HA of A/chicken/Pakistan/SKP-827/16(H9N2), the NA of A/chicken/Pakistan/UDL-01/08(H9N2), and either 6 internal genes of A/chicken/Pakistan/UDL-01/08(H9N2) for virus replication assays, or the 6 internal genes of A/Puerto Rico/08/34(H1N1) for HI and receptor binding assays. The QuickChange Lightning Kit (Agilent) was used in site-directed mutagenesis for residues 180, 134, 148 and 189. For an N-linked glycosylation motif to be introduced for residue 148, amino acid residue 150 needed to be changed from N to T. Amino acid residues 134 and 189 required substitutions S to N, and D to N, respectively. The HA and NA genes for all virus stocks were Sanger sequenced at Source BioScience (Cambridge, UK).

### Virus purification

Virus from allantoic fluid was concentrated and purified for use in biolayer interferometry, western blot and vaccine preparation. The purification of a single virus utilized 450 ml of infectious allantoic fluid from embryonated hens’ eggs. Clarified allantoic fluid was concentrated by ultracentrifugation at 135,000 × g at 4°C for a total of 4 h (2 separate runs of 2 h each) using a Sorvall^TM^ WX90 Ultracentrifuge. Concentrated virus was then homogenized using a glass homogenizer then spun at 1700 × g at 4°C for 5 min using a low-speed centrifuge. Virus supernatant was purified using a density gradient in 30–60% continuous sucrose at 135,000 × g at 4°C for 2 h. A virus-containing fraction within the density gradient was recovered, re-suspended in PBS and pelleted again at 135,000 × g at 4°C for 2 h using the ultracentrifuge. Purified virus was re-suspended in PBS with 0.01% Sodium Azide, aliquoted and stored at 4°C for subsequent use or at −80°C for future use. The concentration of purified virus was determined by enzyme-linked immunosorbent assay (ELISA) by targeting the influenza nucleoprotein (NP) using anti-NP monoclonal antibodies. ELISA assays of purified viruses studied here were conducted alongside ELISA assays of a reference virus of known concentration that was subsequently used to make a standard curve. Concentration of virus was expressed in picomolar (pM) for octet use [19].

### Biolayer interferometry

The receptor binding phenotype of viruses was determined by biolayer interferometry using an Octet RED biolayer interferometer (Pall ForteBio) as previously described [19]. Purified virus and biotinylated receptor analogues α2,6-sialyllactosamine(6SLN), α-2,3 sialyllactosamine(3SLN) and Neu5Ac α-2,3 Gal β1-4(6-HSO3)GlcNAc (3SLN(6su)) (Lectinity Holdings) were used in binding assays. Virus was diluted to 100 pM in HBS-EP buffer (GE Healthcare) containing 10 µM oseltamivir carboxylate (Roche) and 10 µM zanamavir (GSK). Receptor analogues were immobilized onto biosensors coated in streptavidin (Pall ForteBio) at concentrations between 0.01 and 0.5 µg/ml. Binding of virus to receptor analogues was measured at 20°C for 30 min during an association step in the Octet. The Octet read-out of virus binding at the end of the association step was normalized to fractional saturation and plotted as a function of sugar loading. Fractional saturation curves were fitted by a variation of the Hill equation as described previously and used to determine relative *K_D_* values [20,21].

### Virus replication kinetics

*In vitro* virus replication kinetics were studied in MDCK and CK cells with virus at a MOI of 0.001 and 0.01, respectively. Virus was inoculated in triplicate for 1 h prior to washing with PBS and overlaying with virus growth medium (DMEM, FBS, 2 μg/ml tosyl phenylalanyl chloromethyl ketone (TPCK)-treated trypsin) for MDCK cells and CK media for CK cells (as above; no TPCK-treated trypsin added). Culture supernatants were taken at 12, 24, 48 and 72 h post-infection and titrated by plaque assay in MDCK cells [22].

### Haemagglutination inhibition

The hemagglutination assay was first used to determine virus titre and then virus was diluted to 4 HA units and used in haemagglutination inhibition (HI) assays as described previously [22]. Assays were conducted with 1% chicken red blood cells (RBCs) diluted in PBS and HI titers were expressed as the reciprocal of the highest serum dilution at which haemagglutination was completely inhibited.

### SDS-PAGE and western blot

SDS-PAGE and western blot were performed using purified virus to investigate band-shifting as a result of N-linked glycosylation. Mini-PROTEAN® TGX^TM^ (7.5%) precast protein gels (Biorad) were used to perform SDS-PAGE gel electrophoresis. Samples were prepared by denaturing at 95°C for 10 min (non-PNGaseF treated virus only) in protein loading buffer, separated by gel electrophoresis at 100 volts, then transferred to a nitrocellulose membrane soaked in Transfer buffer (distilled water, 192 mM glycine, 25 mM tris-base, 10% v/v methanol) for subsequent western blot using a Trans-Blot Turbo Transfer System (Biorad). After protein had transferred from the precast gel to the nitrocellulose membrane, the membrane was blocked with Blocking buffer (PBS with 0.05% Tween 20 and 5% Marvel milk powder) at 4°C for 24 h then probed with primary antibodies (anti-H9N2 (UDL-01/08) mouse monoclonal antibodies; produced in-house) diluted in antibody dilution buffer (PBS-Tween and 2% Marvel milk powder). The membrane was washed 3 times with PBS-Tween (PBS-T; PBS, 0.05% v/v Tween 20) then probed with secondary antibody (LI-COR Biosciences) diluted in antibody dilution buffer. The membrane was again washed three times with PBS-T before protein was visualized using an Odyssey CLX system (LI-COR Biosciences). A prestained SDS-PAGE protein standard was run in parallel to protein samples.

### PNGase F treatment of viruses

To confirm viruses were being glycosylated *in ovo* after the introduction of an N-linked glycosylation site at residues 134, 148 and 189, concentrated virus was deglycosylated using PNGase F (NewEngland Biolabs), according to manufacturer’s instructions. Briefly, 30 µg of virus was combined with 4 µl of Glycoprotein Denaturing Buffer (10x) in a total reaction volume of 40 µl and denatured at 100°C for 10 min, then chilled on ice for 2 min. Denatured virus was mixed with 4 µl of Glycobuffer 2 (10x), 4 µl of 10% NP-40 and 2 µl of PNGase F enzyme and incubated at 37°C for 18 h. Deglycosylated virus was mixed with protein loading buffer and loaded directly into a gel for SDS-PAGE and western blot.

### Raising polyclonal antisera from chickens

Chicken polyclonal antisera raised against UDL-01/08 virus was generated previously [23]. Chicken polyclonal antisera against SKP-827/16 virus was raised for this study using virus inactivated with 0.1% (v/v) β-propiolactone. Virus inactivation was confirmed by three sequential passages in embryonated hen’s eggs. Inactivated virus was then concentrated by ultracentrifugation at 135,000 × g for 2 h. Concentrated inactivated virus was titrated by HA assay and mixed with oil emersion adjuvant (Montanide; Seppic) at a ratio of 7:3, adjuvant:virus. Three-day-old SPF chickens were inoculated via the subcutaneous route with 1024 HAU of inactivated virus, boosted 10 days later and bled at 18, 25 and 38 days post-inoculation.

## Ethics statement

The use of animals to produce polyclonal antisera was conducted under the guidance and regulations of European and United Kingdom Home Office regulations under project license number P68D44CF. The ethics committee at The Pirbright Institute has approved and reviewed all work involving animals.

## Results

### N-linked glycosylation optimizes receptor-binding in a low or high avidity background

**Figure 1.**
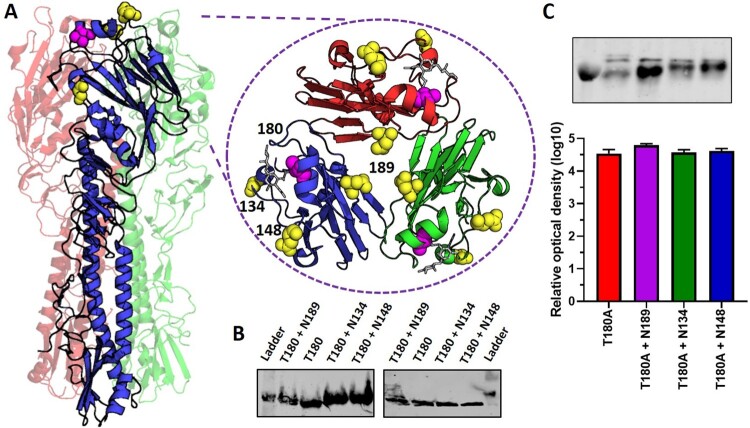
Location of adsorptive mutation and N-linked glycans in H9HA crystal structure. (A) The H9HA homotrimer with each monomer shown as a blue, green or red cartoon. Amino acid residues studied here are shown as spheres; in yellow are asparagine amino acids that become N-linked glycosylated after the introduction of the N-linked glycosylation motif. In magenta is amino acid residue 180 situated in the receptor binding pocket. A top-down blow-up image of the HA1 head is provided with residues 180, 134, 148 and 189 labelled. A sialic acid analog as white sticks occupying the receptor binding pocket is shown. Crystal structure of avian H9 hemagglutinin PDB ID:1JSH [33] and drawn in PyMol [34]. (B) Western blot to confirm N-linked glycosylation at indicated sites on SKP-827/16 T180 virus. Virus purified from allantoic fluid was run on a 7.5% bis-tris SDS-PAGE gel under reducing conditions. Western blot analysis was conducted using anti-H9 mouse monoclonal antibodies. The left panel is untreated virus, the right panel is virus treated with PNGase F. The ladder shows a band at 50 kDa. (C) Western blot analysis was conducted using chicken anti-H9 antisera to detect NP (top band) and HA (bottom) in a single blot. Blot densities were analysed with ImageJ, normalized to the level of detected NP then compared using one-way ANOVA with multiple comparisons. Western blots were repeated three times.

Upon growing and Sanger sequencing virus stocks of the adsorptive mutant SKP-827/16 T180, it was found that on occasion an additional amino acid substitution arose at the consensus level at HA residue 189 whereby aspartate (D) was substituted for asparagine (N). Interestingly, this substitution introduced an N-linked glycosylation motif to the HA protein. As it has been established that glycosylation can compensate for antibody escape fitness costs in other viruses, we therefore wanted to investigate the role of N-linked glycosylation in a H9N2 virus with various adsorptive mutations. To this end we expanded our study to include two additional residues prone to mutations that introduce an N-linked glycosylation motif to the HA protein and which impact virus replication in various *in vitro* and *in vivo* models (Figure 1(A)) [13,24–26]. Thus, we generated reassortant viruses containing A/chicken/Pakistan/SKP-827/2016 (SKP-827/16) HA, A/chicken/Pakistan/UDL-01/2008 (UDL-01/08) NA and the remaining genes of either A/Puerto Rico/8/1934 (PR8) when conducting biolayer interferometry and serology assays, or UDL-01/08 when studying virus replication kinetics. We then conducted site-directed mutagenesis on the SKP-827/16 HA gene to introduce potential glycosylation sites at positions 134, 148 or 189 (single glycan mutants only; Table S1). We further mutated our panel of glycan mutants such that they carried either A, T or V at position 180 (the natural occurrence of these amino acid residues at HA position 180 in H9N2 viruses is 26.9%, 69.67% and 2.32%, respectively). Thus, a panel of viruses with varying degrees of receptor binding avidity were made that had one of three sites potentially glycosylated: 134, 148 or 189. We confirmed glycosylation by observing decreased protein migration rates of SKP-827/16 HAs with extra glycosylation sites compared to the wild type HA on a western blot (Figure 1(B)). Viruses were further treated with PNGase F, which removes N-linked glycosylation, and all showed equivalent protein migration rate of HA, indicating the band shifts in the non-treated viruses was due to differences in glycosylation (Figure 1(B)). Western blot was also used to determine the expression of HA was not directly impacted by the mutations studied here, as shown by densitometry analysis of HA; NP was used for standardization (Figure 1(C)). All virus stocks were Sanger sequenced to confirm no additional mutations arose at the consensus level.

### The addition of N-linked glycans reduces receptor-binding avidity

**Figure 2.**
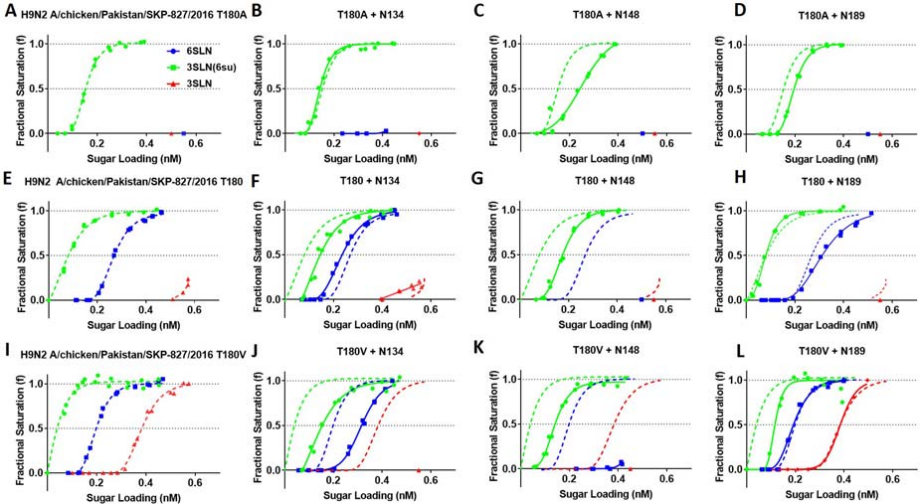
The impact of N-linked glycans on receptor binding phenotype of SKP-827/16 viruses. The impact on receptor binding phenotype of N-linked glycans at amino acid residues 134 (B, F, J), 148 (C, G, K) and 189 (D, H, L) are shown as solid green, blue and red lines. The dashed green, blue and red lines represent the receptor binding phenotype of non-glycosylated parent virus. Parent non-glycosylated virus is shown in panels A, E and I. Green lines represent binding to the receptor analog 3SLN(6Su), blue lines are 6SLN, and red lines are 3SLN.

To determine the impact of N-linked glycans on receptor-binding in three different avidity backgrounds (A, T or V180) we conducted biolayer interferometry. In the low avidity SKP-827/16 T180A virus background, N134 had a negligible effect on binding to 3SLN(6Su), while N148 and N189 both reduced binding avidity towards 3SLN(6Su) (Figure 2(B,C,D)). This reduction in avidity was greater for N148 than N189. In the SKP-827/16 T180 HA background, the addition of glycans at residues 134 and 148 led to reduced binding avidity for the 3SLN(6Su) receptor analog (Figure 2(F,G)). N148 completely ablated 6SLN and 3SLN binding, whereas N189 slightly reduced 6SLN binding but ablated 3SLN binding (Figure 2(G,H)). Interestingly, N134 slightly increased binding avidity towards the 6SLN and 3SLN receptor analogues (Figure 2(B)). Finally, in the highest avidity SKP-827/16 T180 V background, N148 was again shown to completely ablate binding to 6SLN and 3SLN while reducing binding towards 3SLN(6Su) (Figure 2(K)). The addition of N189 had a varied effect whereby it reduced binding avidity to 3SLN(6Su) in the T180A and T180 V backgrounds, had a negligible effect on 3SLN binding and slightly reduced binding avidity to 6SLN in the T180 background (Figure 2(D,H,L)). The addition of N134 reduced binding towards both 3SLN(6Su) and 6SLN while also ablating binding to 3SLN (Figure 2(J)). Interestingly, there was a general trend whereby glycosylation at residue 148 always lowered binding avidity to all receptor analogues while glycosylation at residues 134 and 189 had more context dependent effects, for example N134 in the T180 background facilitated a slight increase in binding avidity to 6SLN and 3SLN. See estimated dissociation constants (KD) provided in Table S2 for quantitative representation of receptor-binding profiles.

### The addition of N-linked glycans optimizes virus replication kinetics

**Figure 3.**
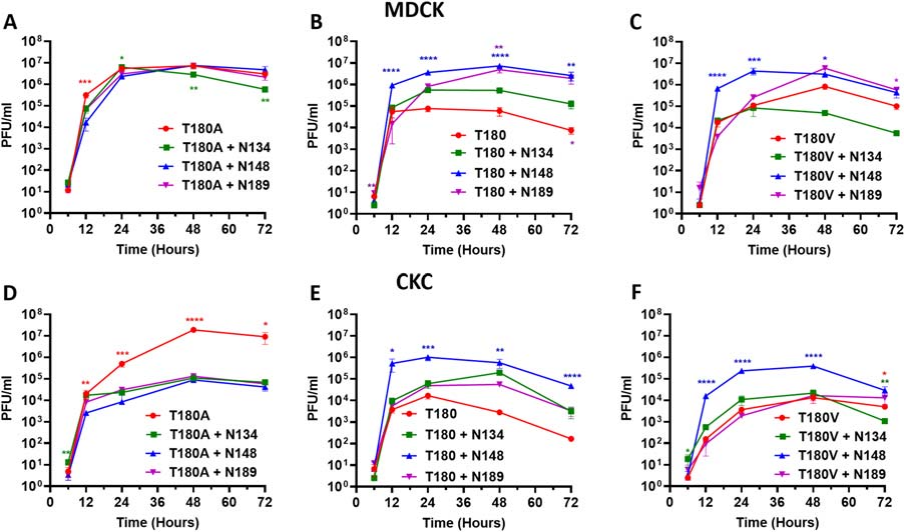
Virus replication kinetics in MDCK and CK cells. (A, B, C) Replication of high and low avidity viruses in MDCK cells after inoculation at a MOI of 0.001. (D, E, F) Replication of high and low avidity viruses in CK cells after inoculation at a MOI of 0.01. Red lines indicate non-glycosylated virus, green lines are virus with N134, blue lines are virus with N148 and purple lines are virus with N189. Virus supernatants were titrated by plaque assay in MDCK cells using culture supernatants harvested at 6, 12, 24, 48 and 72 h post-inoculation. One-way ANOVA with multiple comparisons was used to compare virus titres from each time point. Coloured asterisk next to a same-colour curve indicates that that virus has a significantly different titre to the remaining viruses. Coloured asterisk next to a different-colour curve indicates a statistical difference in titre between that colour and non-glycosylated virus (** equals *P* < 0.01, *** equals *P* < 0.001, **** equals *P* < 0.0001).

Next, we wanted to determine the impact that additional N-linked glycans has on virus replication kinetics in the various avidity backgrounds. We investigated virus replication in MDCK cells as a mammalian *in vitro* model and primary CK cells as an avian *in vitro* model. In agreement with previous data, non-glycosylated high avidity viruses with T or V180 were attenuated compared to virus with low avidity T180A [11] (from hereon, glycosylated and non-glycosylated viruses refers to those viruses which have had N-linked glycosylation motifs at sites 134, 148 and 189 introduced or not through mutagenesis; viruses still contain their naturally occurring N-linked glycosylation motifs at sites other than 134, 148 and 189). However, in the low avidity background of SKP-827/16 T180A, the addition of N-linked glycans led to attenuation, although the difference in viral titers between glycosylated and non-glycosylated virus was greater in CK cells (Figure 3(A,D)). The addition of N148 to the SKP-827/16 T180 virus facilitated significantly higher viral titers at all time points beyond 12 h in both MDCK and CK cells. Similarly, the addition of N189, as seen to arise spontaneously upon replication of this virus in eggs, led to significantly higher viral titers at 6, 48 and 72 hpi in MDCKs only (Figure 3(B,E)). There was a trend of increased virus titres for virus with N134 in MDCK cells however this was not statistically significant (Figure 3(B)). Finally, in the high avidity SKP-827/16 T180 V background virus, the addition of N-linked glycans was similar to their effect in SKP-827/16 T180 virus, such that N148 facilitated significantly greater viral titers in MDCK and CK cells (Figure 3(C,F)). However, in CK cells addition of N-linked glycans at residues 134 and 189 let to comparable virus replication kinetics with non-glycosylated SKP-827/16 T180 V. These effects corroborated well with MDCK plaque phenotype such that the addition of N-linked glycans to virus with T180A lead to significantly smaller plaques, and the addition of N148 to virus with T180 V lead to significantly larger plaques (Figure S1). From these data, we can see that in general, in a low avidity background, glycosylation was detrimental to virus replication, whereas in a high avidity attenuated background, glycosylation enhanced virus replication, especially at position 148.

### Antigenicity is impacted by the interaction between adsorptive mutations and N-linked glycosylation

**Figure 4.**
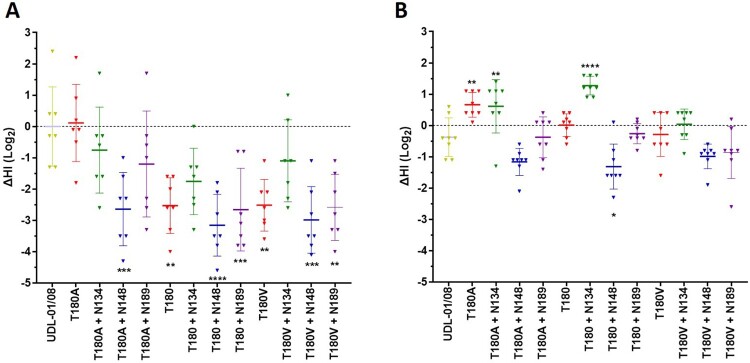
Haemagglutinin inhibition assays were conducted using H9N2 viruses and polyclonal chicken antisera raised against UDL-01/08 or SKP-827/16 virus. (A) HI assays conducted using UDL-01/08 antisera. The mean HI titre from UDL-01/08 virus was normalized to 0 and used to compare antigenic distance of all other tested viruses. (B) HI assays conducted using SKP-827/16 antisera. The mean HI titre from SKP-827/16 virus was normalized to 0 and used to compare antigenic distance as above. Each triangle represents the HI titre from an individual chicken**.** (A) One-way ANOVA was used to compare UDL-01/08 virus with all other viruses, and (B) one-way ANOVA was used to compare SKP-827/16 virus with all other viruses (*** equals *P* < 0.001, **** equals *P* < 0.0001).

As both avidity and N-linked glycosylation can significantly impact antigenicity [10,13,15] we wanted to determine their impact on antigenicity when taken in combination. In the first instance, we utilized chicken polyclonal antisera raised against UDL-01/08 virus (which naturally has A180 and is therefore comparable to SKP-827/16 T180A) in HI assays against our panel of glycosylated and non-glycosylated viruses. Indeed, SKP-827/16 T180A and UDL-01/08 had comparable HI titers against UDL-01/08 antisera (Figure 4(A)), while non-glycosylated SKP-827/16 virus with T180 and T180 V had significantly lower HI titers by up to 4-fold. The addition of N148 to both avidity backgrounds (T and V180) also facilitated significantly lower HI titers that were up to 4-fold lower (Figure 4(A)). Interestingly, the addition of N189 to the SKP-827/16 T180 and T180 V background viruses maintained significantly lower HI titers, and given that N189 in the background of T180A did not have a similar effect, it is possible that the impact of N189 on receptor binding avidity was not sufficient to counteract the enhanced avidity facilitated by T180 and T180 V with respect to antigenicity (Figure 4(A)). Next, we raised polyclonal chicken antisera against non-glycosylated SKP-827/16 T180 virus and conducted HI assays as before. These anti-high avidity virus antisera could reciprocate homologous-like HI titres for glycosylated and non-glycosylated T180, T180A and T180 V viruses in most cases (Figure 4(B)). The HI titres for all viruses with N148 showed statistically significant reductions compared to the homologous titre (Figure 4(B)). Interestingly, the SKP-827/16 T180 antisera had up to two-fold greater HI titre against virus with N134 compared to homologous non-glycosylated SKP-827/16 T180 virus. In the case of SKP-827/16 T180 and T180A but not T180 V with N134 this was significantly enhanced (Figure 4(B)). Taken together, these data show that antisera raised against a low avidity virus has reduced cross-reactivity against closely related high avidity variant viruses or viruses with additional N-linked glycans. However, antisera raised against a high avidity virus generally had greater cross-reactivity against those low avidity variants that did not have the additional glycan at residue 148.

## Discussion

Numerous studies model selection of a virus that can overcome immune pressure and retain “fitness” in a linear fashion [8,15,27–29]. This may seem counterintuitive when considering that viruses co-circulate in their host population as a genetically diverse population and could potentially evolve in numerous different directions. However, evidence suggests that a selective pressure, such as immunity, drives the selection of a single major evolutionary tree [30]. This maintenance of a single evolutionary tree supports the linear fashion of virus selection hypothesis and highlights the importance of understanding the sequence of viral changes involved in staying ahead of immune pressure and being suitably “fit”.

Here we demonstrate the importance of optimizing receptor binding avidity in H9N2 viruses. We confirm that in the background of the SKP-827/16 HA, replication of high avidity non-glycosylated viruses, including viruses carrying T180 and T180 V, there was attenuated replication kinetics in MDCK and CK cells, as shown before [11]. It is reasonable, therefore, to assume these attenuated viruses would benefit from compensatory mutations; the addition of N-linked glycans as compensatory mutations was studied for two reasons. First, growing wild type SKP-827/16 virus that naturally contains T180 led to the addition of HA D189N which glycosylated residue 189, indicating proven biological relevance to studying N-linked glycans. Second, N-linked glycosylation has been shown to be important in various models of antigenic/viral fitness evolution involving *in vitro* and *in vivo* studies [13,14]. We showed that in all cases where an N-linked glycan was added to the high avidity virus containing T180, virus replicated to greater titres compared to non-glycosylated T180, and N148 had the largest impact in both MDCK and CK cells. In fact, the addition of N148 to virus with any avidity background led to reduced binding avidity to all tested receptor analogues and a significant impact on virus replication apart from T180A viruses in MDCK cells. Glycosylation at residue 148 also facilitated reduced haemagglutination inhibition regardless of antisera used. Indeed, N148 facilitated a receptor binding profile that resembled low avidity non-glycosylated T180A virus suggesting that the significant reduction in HI titre was because of the antigenic mechanism whereby an N-linked glycan occludes the receptor binding site. We could infer that amino acid residue 148 is situated at the most conducive point to impede both HA binding with sialic acid and antibody binding to the RBS. On the other hand, glycosylation of residues 134 and 189 had comparably less impact on receptor binding and seemed to have a context dependent effect. In virus with T180, N134 increased binding to both non-sulphated receptor analogues but reduced binding to sulphated 3SLN. In virus with V180, N134 reduced binding to all tested analogues. With regards to antigenicity, when a glycan is added to a low avidity virus, occlusion of the receptor binding site by a glycan is causing a reduction in HI titre. However, it is difficult to discern whether antigenicity is being impacted by avidity or occlusion by a glycan in the case of high avidity virus with N134 or N189, although in the case of N189 it is most likely due to avidity because the impact on receptor binding is comparably slight. The enhanced impact of N148 compared to N134 and N189 may also be reflected by the prevalence of these glycans in nature; 2.6%, 0.1% and 0.1%, respectively.

Human influenza viruses offer an excellent example of linear evolution involving the addition of N-linked glycans over time. A study by Altman *et al.* showed that seasonal H1 and H3 HAs accumulated N-linked glycans at 5-to-7-year intervals, totalling 5 and 7 glycans for H1 and H3 HAs, respectively, before a functional limit was reached, by which time glycans were swapped between residues at 2-fold-longer intervals [31]. In contrast, seasonal H2 HAs experienced no change in N-linked glycan status during their ∼11 year circulation (maintaining a single N-linked glycan on the HA head domain). Although the addition of N-linked glycans to H2 HA has been demonstrated *in vitro* during immune escape, glycosylation has been shown to significantly reduce cell fusion and receptor binding functionality [32]. Taken together these data suggest that N-linked glycosylation has a role in the persistence of seasonal H1, H2 and H3 viruses, and our data here suggest the same could apply to avian influenza viruses.

It is particularly interesting that antisera raised against the high avidity virus (T180) could only poorly inhibit the haemagglutination ability of SKP-827/16 T180 virus carrying an N-linked glycan at residue 148. This suggests N-linked glycosylation at residue 148 trumps avidity in this case. Furthermore, given the improved replication kinetics of this virus relative to non-glycosylated T180 virus it is possible that such a virus would persist – it can evade immune pressure even from high avidity antisera while retaining fitness.

The use of mutations to modulate avidity or introduce an N-linked glycan are interchangeable during immune escape and compensatory mutation. More specifically, an N-linked glycan can be used to escape immunity, or it can be used to achieve viral fitness, likewise with avidity mutations. In the data presented here, it is plausible that an H9N2 virus could escape immunity through addition of an N-linked glycan at residues 134, 148 or 189 and subsequently achieve viral fitness with a mutation that modulated avidity such as with substitutions A180 T/V, or *vice versa*. Importantly, a virus containing T180 and a glycan at residue 134 or 189 still has the capacity to bind human-like receptors. Thus, we have a virus that escaped immunity, recovered viral fitness after a period of attenuation and then enhanced its capacity for zoonotic infection. Taken together these data highlight how viral fitness is a fine balance to be maintained and tuned by choice mutations, which can often be pleomorphic. To conclude, avidity and glycosylation can act together to allow efficient immune escape whilst also potentially changing receptor preference and promoting viral fitness.

## Supplementary Material

TEMI_2020_0065.R2_Table_S1_and_Table_S2.docx

Figure_S1.png
