# Supplementary material for: Adsorptive mutation and N-linked glycosylation modulate influenza virus antigenicity and fitness
Source: Emerg Microbes Infect. 2020 Dec 14;9(1):2622–31. doi: 10.1080/22221751.2020.1850180 (PMC7738305; doi:10.1080/22221751.2020.1850180)
Supplement: TEMI_2020_0065.R2_Table_S1_and_Table_S2.docx [file TEMI_A_1850180_SM9656.docx]

**Table S1. List of viruses made with amino acid substitutions in the HA protein**

| **Virus backbone** | **Amino acid substitution** | **Glycosylated residue** | **New virus name** |
| --- | --- | --- | --- |
| **SKP-827/16 T180A** | - | - | - |
| **SKP-827/16 T180A** | S134N | 134 | T180A + N134 |
| **SKP-827/16 T180A** | N150T | 148 | T180A + N148 |
| **SKP-827/16 T180A** | D189N | 189 | T180A + N189 |
| **SKP-827/16 T180** | - | - | - |
| **SKP-827/16 T180** | S134N | 134 | T180 + N134 |
| **SKP-827/16 T180** | N150T | 148 | T180 + N148 |
| **SKP-827/16 T180** | D189N | 189 | T180 + N189 |
| **SKP-827/16 T180V** | - | - | - |
| **SKP-827/16 T180V** | S134N | 134 | T180V + N134 |
| **SKP-827/16 T180V** | N150T | 148 | T180V + N148 |
| **SKP-827/16 T180V** | D189N | 189 | T180V + N189 |

**Table S2. Estimated dissociation constants (KD) from biolayer interferometry.**

|  | **Estimated KD (μM)** | | |
| --- | --- | --- | --- |
| **Virus** | **3SLN(6Su)** | **6SLN** | **3SLN** |
| **SKP-827/16 T180A** | 221.37 | - | - |
| **SKP-827/16 T180A+N134** | 132.54 | - | - |
| **SKP-827/16 T180A+N148** | 2068.17 | - | - |
| **SKP-827/16 T180A+N189** | 713.93 | - | - |
| **SKP-827/16 T180** | 8.11 | 2688.57 | - |
| **SKP-827/16 T180+N134** | 143.28 | 1384.60 | - |
| **SKP-827/16 T180+N148** | 405.63 | - | - |
| **SKP-827/16 T180+N189** | 12.34 | 5277.67 | - |
| **SKP-827/16 T180V** | 0.06 | 701.17 | 12945.22 |
| **SKP-827/16 T180V+N134** | 148.89 | 5517.94 | - |
| **SKP-827/16 T180V+N148** | 146.06 | - | - |
| **SKP-827/16 T180V+N189** | 59.78 | 574.81 | 13552.94 |

Values are in 532 μM, - means there was no detectable binding to this analogue although it was tested.
