# Supplementary figures and images for: Adsorptive mutation and N-linked glycosylation modulate influenza virus antigenicity and fitness
Source: Emerg Microbes Infect. 2020 Dec 14;9(1):2622–31. doi: 10.1080/22221751.2020.1850180 (PMC7738305; doi:10.1080/22221751.2020.1850180)

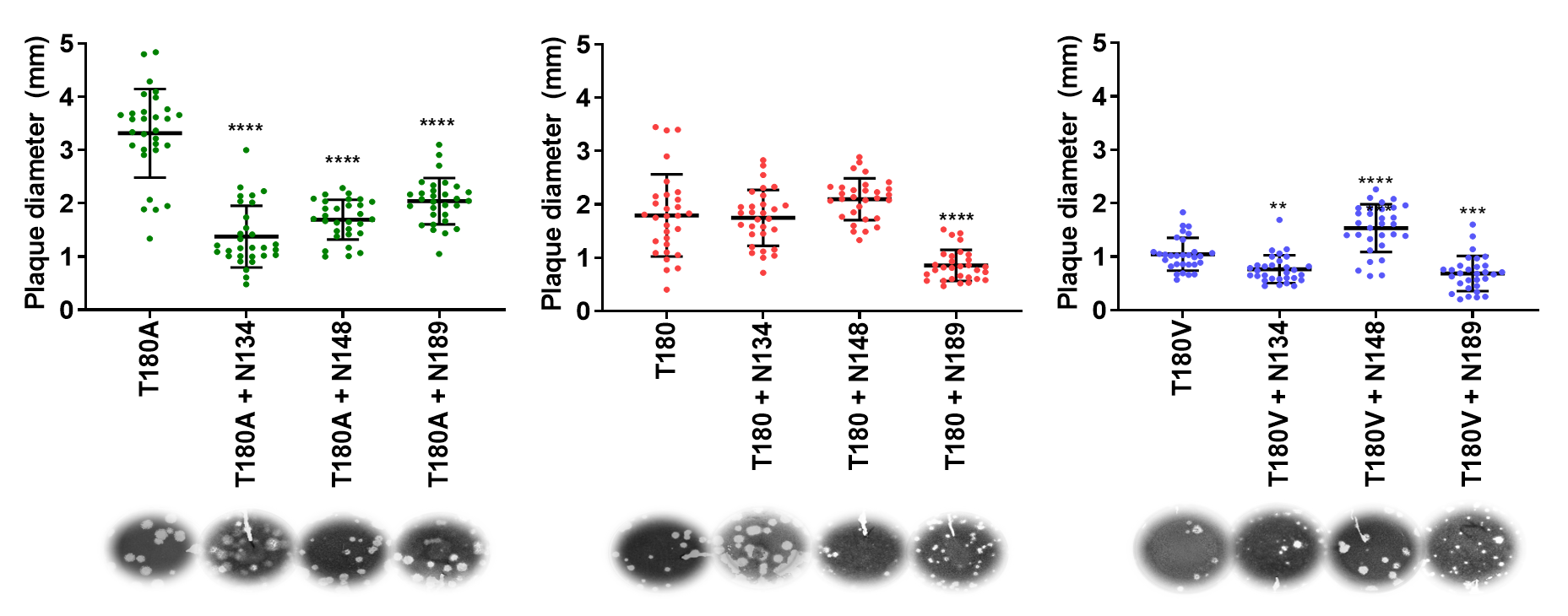

Supplement: Figure_S1.png [file TEMI_A_1850180_SM9655.png]
